# 24‐Month HIV‐free survival among HIV‐exposed Infants in Lesotho: the PEAWIL cohort study

**DOI:** 10.1002/jia2.25648

**Published:** 2020-12-12

**Authors:** Vincent J Tukei, Rhoderick Machekano, Michelle M Gill, Appolinaire Tiam, Majoalane Mokone, Anthony Isavwa, Malijane Nyabela, Tsietso Mots’oane, Seipati Nchephe, Mosilinyane Letsie, Seble G Kassaye, Laura Guay

**Affiliations:** ^1^ Elizabeth Glaser Pediatric AIDS Foundation Maseru Lesotho; ^2^ Elizabeth Glaser Pediatric AIDS Foundation Washington DC USA; ^3^ Centre for International Health University of Bergen Norway; ^4^ Ministry of Health Maseru Lesotho; ^5^ Department of Medicine Georgetown University School of Medicine Washington DC USA; ^6^ Department of Epidemiology George Washington University Milken Institute School of Public Health Washington DC USA

**Keywords:** human immunodeficiency virus, prevention, mother‐to‐child‐transmission, infant, mortality, Lesotho, HIV‐free survival

## Abstract

**Introduction:**

Following the implementation of the provision of lifelong antiretroviral therapy to all HIV‐positive pregnant or breastfeeding women for prevention of mother‐to‐child transmission (PMTCT) of HIV by the Kingdom of Lesotho in 2013, we assessed the effectiveness of this approach by evaluating 24‐month HIV‐free survival among HIV‐exposed infants (HEIs).

**Methods:**

We conducted a prospective observational cohort study that enrolled HIV‐positive and HIV‐negative pregnant women, with follow‐up of women and their infants for 24 months after delivery. Participant recruitment started in June 2014 and follow‐up ended in September 2018. Trained nurses collected study information through patient interviews and chart abstraction at enrolment and every three to six months thereafter. Maternal HIV testing, infant mortality, HIV transmission and HIV‐free survival rates were computed using Kaplan–Meier estimation. Cox regression hazard models were used to identify factors associated with infant HIV infection and death.

**Results:**

Between June 2014 and February 2016, we enrolled 653 HIV‐positive and 941 HIV‐negative pregnant women. Twenty‐seven HIV‐negative women acquired HIV during follow‐up. Ultimately, 634 liveborn HEI (382 (52%) male, 303 (48%) female, 3 missing) and 839 who remained HIV‐unexposed (HUIs) (409 (49.0%) male, 426 (51.0%) female, 4 missing) were followed; 550 HEIs and 701 HUIs completed the 24‐month follow‐up period. Of 607 (95.7%) HEIs who were tested for HIV at least once during follow‐up, 17 were found to be HIV‐positive. Two (9.5%) of 21 infants born to mothers who acquired HIV infection during follow‐up were HIV‐positive compared to 15 (2.4%) of 613 HEI born to women with known HIV infection. The risk of HIV transmission from HIV‐positive mothers to their infants by 24 months of age was 2.9% (95% CI: 1.8 to 4.7). The estimated 24‐month mortality rate among HEIs was 6.0% (95% CI: 4.4 to 8.2) compared to 3.8% (95% CI: 2.6 to 5.3) among HUIs (Log‐rank *p* = 0.065). HIV‐free survival at 24 months was 91.8% (95% CI: 89.2 to 93.7). Lower maternal age and birth weight were independently associated with increased HIV infection or death of infants.

**Conclusions:**

The implementation of lifelong ART for PMTCT in the Lesotho public health system resulted in low HIV transmission, but survival of HEI remains lower than their HIV uninfected counterparts.

## 1 INTRODUCTION

The HIV epidemic in children is largely due to mother‐to‐child HIV transmission (MTCT). In 2018, the Joint United Nations Program on HIV/AIDS (UNAIDS), estimated that 160,000 new HIV infections occurred among children [1]. Without treatment, approximately one‐half of HIV‐infected children in sub‐Saharan Africa die before their second birthday [2, 3]. Preventing new infections and providing early treatment to infected infants are essential strategies for controlling the HIV epidemic in infants and children [3].

Over the last two decades, strategies for the prevention of mother‐to‐child HIV transmission (PMTCT) have evolved to include maternal use of highly active antiretroviral drugs [4]. The implementation of these PMTCT strategies has led to tremendous success in the prevention of new infections among children [5, 6, 7]. In 2013, the World Health Organization (WHO) released new guidelines on the use of antiretroviral drugs for the treatment of pregnant women and the prevention of HIV infection in infants [8]. These guidelines, initially known as Option B+, recommended lifelong antiretroviral therapy (ART) for all pregnant and breastfeeding women with a six‐week administration of daily nevirapine to the infant.

In settings where this PMTCT strategy is fully implemented, a substantial reduction in new HIV infections has been realized. There is clear evidence showing improved HIV‐free survival for infants tested at six weeks of age [9, 10, 11]; however, there is limited information on the long‐term effectiveness of this strategy among breastfeeding populations in high HIV prevalence settings where infants often continue to be exposed to HIV throughout the first two years of life. In 2013, the Kingdom of Lesotho, with an HIV prevalence of 25.8% among pregnant women, adopted these WHO guidelines.

The PMTCT Program Effectiveness among Women and Infants in Lesotho (PEA‐WIL) study aimed to evaluate the effectiveness of PMTCT service delivery and assess progress towards the virtual elimination of mother‐to‐child HIV transmission (EMTCT) in selected districts of Lesotho [12]. This paper presents findings on 24‐month HIV‐free survival among HEI, factors associated with infant HIV infection and death, and compares survival among HEI and HUI.

## 2 METHODS

### 2.1 Study design

This was a prospective observational cohort study that involved the recruitment and follow‐up of pregnant women attending antenatal care (ANC) clinics at selected health facilities in Lesotho. We recruited HIV‐positive and HIV‐negative pregnant women and followed them during pregnancy up to 24 months postpartum. Infants born to these women were followed along with their mothers for the 2‐year period after delivery.

### 2.2 Study setting

During the study period, health facilities provided routine PMTCT services to all pregnant women as part of general ANC. Pregnant women were offered opt‐out HIV screening followed by ART initiation if the women tested HIV positive. HIV‐negative women received repeat HIV tests at 36 weeks of pregnancy or delivery, and in the postnatal period. Based on the national PMTCT guidelines, the preferred ART regimen was a fixed‐dose combination of tenofovir disoproxil fumarate, lamivudine and efavirenz administered once daily. HIV viral load monitoring was carried out every six months, and women found to have viral load results <1000 copies/mL were considered virally suppressed.

HEIs were initiated on daily nevirapine from birth to six weeks of age and on daily cotrimoxazole from six weeks of age until HIV infection was definitively excluded. Exclusive breastfeeding for the first six months of life was encouraged. To assess HIV infection in the infant, HIV DNA PCR tests were carried out at four and fourteen weeks of infant age. Infants with negative DNA PCR results were subsequently re‐tested using WHO‐recommended rapid antibody tests at nine months, with confirmatory DNA PCR testing if positive. Infants with negative HIV test results at nine months, received a rapid antibody test at 18 months of age or six weeks after cessation of breastfeeding. HIV‐positive infants were initiated on ART.

### 2.3 Study sites and participants

Between June 2014 and February 2016, we consecutively enrolled pregnant women from 14 health facilities (five hospitals and nine health centres) located in three of Lesotho’s ten districts. These health facilities represented all hospitals and a random selection of medium (100 to 200 ANC attendees/year) and high volume (>200 ANC attendees/year) health centres in the three districts.

To be enrolled in the study, pregnant women had to be attending ANC at a study facility, reside within the facility catchment area, and be willing to provide written informed consent. There was no age restriction for study enrolment as young pregnant women in Lesotho are considered emancipated and therefore provided their own consent for participation. Women who temporarily received care from the health facility were excluded. Infants were automatically eligible for enrolment at birth.

Infants were considered HIV exposed if their mothers were found to be HIV positive during pregnancy, delivery or during the breastfeeding period, including infants born to HIV‐negative women who seroconverted during follow‐up.

### 2.4 Study procedures

Study‐specific information was obtained at enrolment, around the time of delivery, and every three months postpartum. Gestational age at enrolment was determined based on the date of the last menstrual period. Trained study nurses interviewed the women and abstracted additional study information from the medical records using structured data collection forms. The nurses reviewed participant medical records and abstracted information on general health and clinical/laboratory history of infants, birth history, hospitalizations, ARV use and toxicity, retention in care, infant feeding practices and infant growth. Relevant information on deliveries or admissions that occurred in non‐study facilities, was extracted from the participant’s medical booklet upon return to the study facility. At every visit, participants were interviewed to assess adherence to ART in the seven days preceding the visits. In addition, we conducted study‐specific HIV DNA PCR testing at birth and at six months, and a final rapid antibody test on all exposed children at 24 months of age.

HIV‐unexposed infants (HUIs) were assessed every three to six months when their mothers returned to the clinics for postnatal care and HIV re‐testing. Women were re‐tested every three months until 12 months postpartum and at 18 and 24 months postpartum. HIV‐negative women who seroconverted during follow‐up were started on ART and their infants tested for possible HIV infection. At every study visit, study nurses collected information on infant feeding practices.

For children who died during follow‐up, mortality information was obtained through chart abstraction at the health facilities. Information on date of death, the primary cause of death and medication given to the child at the time of death was abstracted. For deaths that occurred outside health facilities, parents or caregivers were interviewed to determine events at the time of death.

### 2.5 Statistical analysis

We summarized maternal and infant characteristics at delivery using frequencies and proportions by infant HIV exposure status. Continuous variables were summarized using means (±SD) or medians (range) by exposure status. We also summarized and compared 24‐month infant outcomes using frequencies and proportions with associated 95% confidence intervals. Chi‐square tests were used to test for significant differences between HIV‐exposed and ‐unexposed infants.

For each infant who did not die during follow‐up, we calculated the time at risk of death from birth to the last follow‐up date. For infants who died, their time at risk for death was calculated as the difference between date of birth and date of death. We created an indicator variable for death, with those who did not die censored at the last known follow‐up date. The HIV infection risk time was calculated only among HIV‐exposed infants who had at least one HIV test. For infants who were not infected, the time at risk for HIV infection was calculated as the difference between the date of birth and the date of last HIV‐negative test. For infants who became infected with HIV, their time at risk for HIV infection was calculated as the difference between the date of birth and the date of the first HIV‐positive test. An HIV infection indicator variable was created with infants who were not infected censored at the last HIV‐negative test date. We created an indicator for HIV‐free survival based on either death or HIV infection and the HIV‐free survival time was calculated as the minimum time between being at risk of death and at risk for HIV infection.

We estimated infant mortality, MTCT and HIV‐free survival rates over time using Kaplan Meier estimation. Kaplan Meier graphs were used to describe mortality, infection and survival patterns over time. We used Wilcoxon log‐rank tests to compare the survival curves between HIV‐exposed and unexposed infants.

We estimated the proportion of infants who died or were infected by the different categories of maternal and infant characteristics assessed at delivery. We used univariate Cox regression to identify factors associated with infant death or HIV infection. Factors that were significantly associated with infant death or HIV infection in the unadjusted analyses were included in a multivariate Cox regression model to identify independent factors associated with death or HIV infection. The strength of the association was expressed as adjusted hazard ratios and associated 95% confidence intervals. All statistical analyses were performed using Stata version 15.1 (College Station, TX, USA).

### 2.6 Ethical considerations

This study was approved by the Lesotho National Health Research Ethics Committee, the Baylor College of Medicine Children’s Foundation Lesotho Institutional Review Board (IRB), and the George Washington University IRB. Written informed consent was obtained from the mothers for their own participation and the participation of their infants. All study team members were trained on the protection of human subjects.

## 3 RESULTS

**Figure 1:**
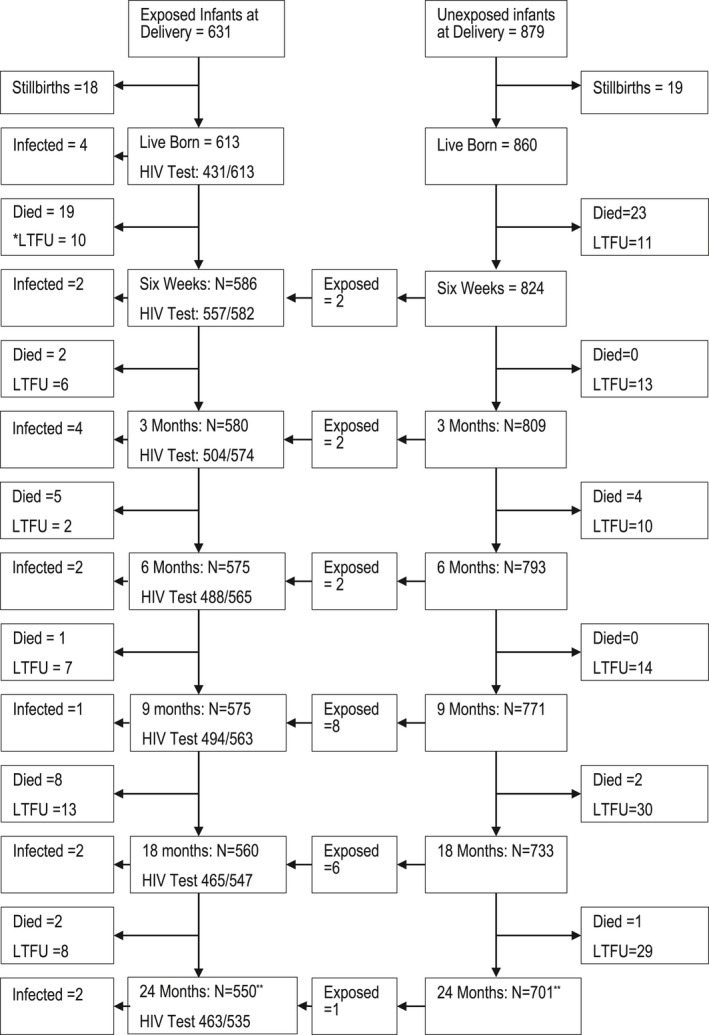
Child follow‐up and HIV testing from birth to 24 months postpartum. *Note:* *LTFU = Loss to follow‐up;** 1 additional child death in each group with unknown age at death.

A total of 1594 pregnant women were enrolled in the study, of which 653 (41.0%) were HIV positive and 941 (59.0%) were HIV negative. By the time of delivery, eight HIV‐negative women had sero‐converted to become HIV positive. Birth information was available for 631 infants that were HIV exposed by delivery (613 (97.1%) liveborn, 18 (2.9%) stillborn) and 879 HIV unexposed infants (860 (97.8%) liveborn, 19 (2.2%) stillborn) (Figure 1). This includes eight sets of HEI twins and 11 sets of HUI twins. During the postpartum follow‐up period, an additional 19 HIV‐negative women with 21 children seroconverted. Altogether, 634 liveborn infants (328 (52.0%) male, 303 (48%) female, 3 missing) were HIV exposed. 24‐month outcomes were available for 588 HEI (550 (93.5%) alive and 38 (6.5%) died) and 733 HUI (701 (95.6%) alive and 32 (4.4%) died) with 87% of eligible HEI having a 24‐month final HIV test done.

Overall, 45.5% of mothers was under age 25 years; of the 19 women who seroconverted after delivery, 84% was under age 25 years (Table 1). The proportion of women who disclosed their HIV status to someone was 74.4%, with 66.7% disclosing to their partner/spouse. At delivery, 96.9% of mothers of HIV‐exposed infants was on ART (median time on ART = 6.6 months; IQR: 4 to 31.6 months). Of 595 HIV‐exposed infants with information on infant antiretroviral prophylaxis, 576 (96.8%) were initiated on nevirapine, with the majority of infants initiating prophylaxis within the first 72 hours of life. About 50% of HIV‐exposed and unexposed infants with information available on breastfeeding practices was exclusively breastfed for the first six months of life. Mixed feeding in the first six months of life was reported in 218/551 (39.6%) HIV‐exposed infants and 337/705 (47.8%) HIV‐unexposed infants.

**Table 1:** Maternal and infant characteristics by child HIV exposure status among liveborn infants

Characteristic	HIV exposed at delivery	HIV exposed during follow‐up	HIV unexposed	Total
	n (%)	n (%)	n (%)	
Characteristics of mothers of liveborn infants				
Number of mothers with delivery information	607	19	830	1456
Age at delivery in years median (range)	29 (17 to 45)	21 (15 to 28)	23 (14 to 45)	25 (21 to 31)
Age group (years)				
14 to 24	158 (26.2%)	16 (84.2%)	487 (58.7%)	661 (45.5%)
25 to 45	446 (73.8%)	3 (15.8%)	343 (41.3%)	792 (54.5%)
Marital status				
Married/living with partner	492 (81.5%)	14 (73.7%)	719 (86.6%)	1225 (84.3)
Never married	70 (11.6%)	4 (21.1%)	106 (12.8%)	180 (12.4)
Divorced/widowed	42 (7.0%)	1 (5.3%)	5 (0.6%)	48 (3.3)
Education				
Primary or less	265 (43.9%)	5 (26.3%)	278 (33.5%)	548 (37.7)
Secondary	202 (33.4%)	10 (52.6%)	314 (37.8%)	526 (36.2)
High school	97 (16.1%)	4 (21.1%)	175 (21.1%)	276 (19.0)
Tertiary	40 (6.6%)	0	63 (7.6%)	103 (7.1)
HIV status disclosure (at any time)				
Disclosed to someone	518 (85.8%)	11 (57.9%)	552 (66.5%)	1081 (74.4)
No disclosure	86 (14.2%)	8 (42.1%)	278 (33.5%)	372 (25.6)
HIV status disclosure to husband/partner				
Disclosed	353 (74.5%)	7 (50.0%)	445 (61.9%)	805 (66.7)
Did not disclose	121 (25.5%)	7 (50.0%)	274 (38.1%)	402 (33.3)
On ART at delivery or during follow‐up				
Yes	588 (96.9%)	13 (61.9%)	NA	
No	19 (3.1%)	8 (38.1%)		
Delivery mode				
Vaginal	511 (84.7%)	13 (68.4%)	717 (86.8%)	1241 (85.7)
Cesarean	92 (15.3%)	6 (31.6%)	109 (13.2%)	207 (14.3)
Viral suppression (during entire follow‐up period)				
Suppressed (<1000 copies/mL)	358 (60.2%)	3 (25.0%)	NA	361 (59.5)
Not suppressed	237 (39.8%)	9 (75.0%)		246 (40.5)
Infant characteristics				
Number of live infant births	613	21	839	1473
Sex				
Male	314 (51.5%)	14 (66.7%)	409 (49.0%)	737 (50.3%)
Female	296 (48.5%)	7 (33.3%)	426 (51.0%)	729 (49.7%)
Infant feeding in first 6 months				
Exclusive breast feeding	279 (52.0%)	6 (40%)	349 (49.5%)	
Exclusive alternatives to breast feeding	47 (8.8%)	1 (6.7%)	19 (2.7%)	
Mixed feeding	210 (39.2%)	8 (53.3%)	337 (47.8%)	
Infant initiated on ARV prophylaxis				
Yes	576 (96.8%)	NA	NA	
No	19 (3.2%)			
ARV prophylaxis initiated within 72 hours				
Yes	560 (94.8%)	NA	NA	
No	31 (5.2%)			
Birth weight in kg	3.03 (0.52)	3.02 (0.69)	3.12 (0.53)	3.08 (0.53)
mean (SD)				
Birth weight category				
<2.5 kg	59 (10.4%)	4 (23.5%)	65 (8.3%)	128 (9.3)
>2.5 kg	511 (89.6%)	13 (76.5%)	718 (91.7%)	1242 (90.7)
Infant maturity at birth				
Term	568 (93.3%)	20 (100%)	803 (96.6%)	1391 (95.3)
Pre‐term^a^	41 (6.7%)	0	28 (3.4%)	69 (4.7)

^a^ Preterm = Born alive before 37 completed weeks of gestation.

During the 24‐month follow‐up period, 38 (6.0%) HIV‐exposed infants died compared to 31 (3.7%) HIV‐unexposed infants (chi‐square *p* = 0.039) (Table 2).

**Table 2:** Infant outcomes by final HIV exposure status

Outcome	HIV exposed	HIV unexposed	*p*‐value
	N (%)	N (%)	
Total live births	634	839	
Died	38 (6.0%)	31 (3.7%)	0.039
HIV infected	17/607 (2.8%)	0	–
Died or HIV infected	52 (8.2%)	31 (3.7%)	<0.001
HIV‐free survival^a^	581 (91.8%)	806 (96.3%)	

^a^ HIV‐free survival could not be determined for 3 infants (1 HEI and 2 HUI) with missing data

The diagnosis preceding death is as shown in Table 3 below.

**Table 3:** Diagnosis preceding death of infants during follow‐up

Diagnosis	HIV exposed infants	HIV unexposed infants	Total
Acute respiratory tract infection	7	5	12
Diarrhoea and vomiting	5	2	7
Neonatal sepsis	5	2	7
Birth asphyxia	2	8	10
Severe acute malnutrition	2	0	2
Congenital abnormalities	1	1	2
Foreign body aspiration	1	1	2
Vitamin K deficiency bleeding	1	0	1
Accidental drowning	0	1	1
Intestinal obstruction	0	2	2
Unknown	14	9	23
Total	38	31	69

A total of 607 (95.7%) HIV‐exposed infants were tested for HIV at least once during follow‐up. Of these, 17 (2.8%) were HIV infected. Four of the 17 infants were diagnosed at birth (in utero infection rate, 0.7%). Two (9.5%) of the 21 infants born to mothers who acquired HIV infection during follow‐up were HIV infected compared to 15 (2.4%) of 613 HEI tested at birth who were born to women with known HIV infection. All HIV‐positive infants were put on ART. Table 4 shows the estimated mortality, HIV transmission and HIV‐free survival rates over time by exposure status. The estimated 24‐month mortality rate among HIV‐exposed infants was 6.0% (95% CI: 4.4 to 8.2) compared to 3.8% (95% CI: 2.6 to 5.3) among HIV‐unexposed infants (Log‐rank *p* = 0.065). The overall estimated risk of HIV transmission from HIV‐positive mothers to their infants was 2.9% (95% CI: 1.8 to 4.7).

**Table 4:** Estimated child mortality, infection and HIV‐free survival rates by HIV exposure status among liveborn infants

Time	Mortality rate		HIV transmission rate % [95% CI]	HIV‐Free Survival rate Survival rate	
	HIV exposed % [95% CI]	HIV unexposed % [95% CI]		HIV exposed % [95% CI]	HIV unexposed % [95% CI]
6 weeks	2.4 [1.5 to 4.0]	2.7 [1.8 to 4.1]	0.6 [0.3 to 1.8]	96.8 [95.1 to 97.9]	97.3 [95.9 to 98.2]
6 months	4.1 [2.8 to 6.0]	3.3 [2.3 to 4.8]	1.9 [1.0 to 3.4]	94.5 [92.3 to 96.0]	96.7 [95.2 to 97.7]
12 months	4.9 [3.5 to 7.0]	3.5 [2.4 to 5.0]	2.1 [1.2 to 3.6]	92.7 [90.4 to 94.5]	96.5 [95.0 to 97.6]
18 months	5.6 [4.1 to 7.8]	3.8 [2.6 to 5.3]	2.2 [1.3 to 3.8]	92.6 [90.1 to 94.4]	96.2 [94.7 to 97.4]
24 months	6.0 [4.4 to 8.2]	3.8 [2.6 to 5.3]	2.9 [1.8 to 4.7]	91.8 [89.2 to 93.7]	96.2 [94.7 to 97.4]
Log rank *p*	0.065		–	<0.001	

**Figure 2:**
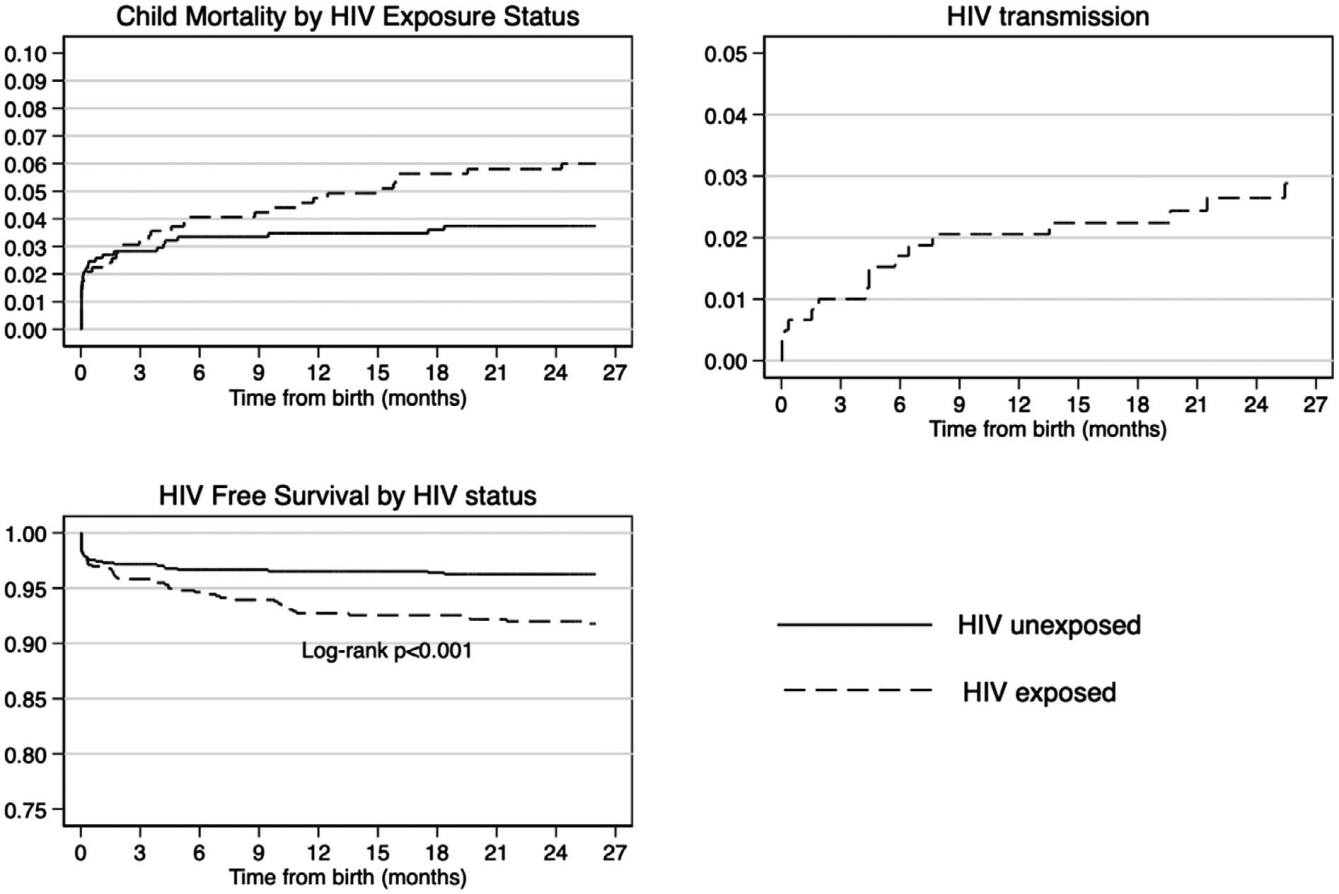
Kaplan‐Meier graphs of child mortality, HIV transmission and HIV‐free survival by HIV exposure status among liveborn infants.

Figure 2 displays Kaplan–Meier curves of mortality, HIV transmission and HIV‐free survival for the infants by HIV exposure status. Mortality is high during the first two months of life for both HIV‐exposed and HIV‐unexposed infants but thereafter, mortality among HEIs is markedly higher than mortality in HUIs. The rate of in utero infection was low, 0.7%, with most infection occurring during the postnatal period through age 12 months.

In unadjusted models, younger maternal age, lack of viral suppression, lower birth weight, and prematurity were independently associated with increased HIV infection or death of HEI (Table 5). Marital status, maternal education, disclosure of HIV status to someone else or husband, being on ART at delivery, delivery mode, type of infant feeding, sex of child and ARV prophylaxis were not associated with the hazard of HIV infection or death.

**Table 5:** Maternal and infant factors associated with infant’s HIV infection or death in exposed infants (N = 634)

Characteristic	Total (n)	Infected or died, n (%)	Unadjusted HR [95% CI]	Adjusted HR [95% CI]	*p*‐value
Maternal characteristics					
Mother’s age (years)					
14 to 24	174	25 (14.4%)	2.53 [1.45 to 4.44]	2.51 [1.29 to 4.87]	0.007
25 to 45	448	26 (5.8%)	1	1	
Marital status					
Married/living with partner	505	38 (7.5%)	1		
Never married	74	10 (13.5%)	1.75 [0.85 to 3.64]		
Divorced/widowed	43	3 (7.0%)	0.94 [0.29 to 3.04]		
Education					
Primary or less	269	26 (9.7%)	1		
Secondary	212	15 (7.1%)	0.69 [0.36 to 1.33]		
High school	101	8 (7.9%)	0.81 [0.37 to 1.81]		
Tertiary	40	2 (5.0%)	0.54 [0.13 to 2.28]		
HIV status disclosure					
Disclosed to someone	528	39 (7.4%)	1		
No disclosure	94	12 (12.8%)	1.89 [0.99 to 3.63]		
Disclosure to husband/partner					
Disclosed	400	27 (6.8%)	1	1	
Did not disclose	222	24 (10.8%)	1.71 [0.98 to 3.0]		
On ART at delivery					
Yes	587	43 (7.3%)	1		
No	18	3 (16.7%)	1.21 [0.17 to 8.79]		
Viral suppression					
Suppressed	361	19 (5.3%)	1	1	
Not Suppressed	245	25 (10.2%)	2.05 [1.13 to 3.73]	1.81 [0.92 to 3.56]	0.089
Delivery mode					
Vaginal	524	39 (7.4%)	1		
Cesarean	98	10 (10.2%)	1.36 [0.68 to 2.72]		
Infant characteristics					
Sex					
Male	324	19 (5.9%)	1		0.08
Female	297	29 (9.8%)	1.68 [0.94 to 2.99]		
Infant feeding in first 6 months of life					
Exclusive breast feeding	280	19 (6.8%)	1		
Exclusive formula feeding	47	1 (2.1%)	0.29 [0.04 to 2.17]		
Mixed feeding	216	10 (4.6%)	0.62 [0.29 to 1.34]		
Initiated ARV prophylaxis					
Yes	569	35 (6.2%)	–		
No	19	0			
ARV prophylaxis within 72 hours					
Yes	554	34 (6.1%)	1		
No	30	1 (3.3%)	0.53 [0.07 to 3.84]		
Birth weight (per 100 grams increase)	567	37	0.88 [0.84 to 0.92]	0.93 [0.86 to 0.99]	0.04
Maturity at birth					
Term	581	39 (6.7%)	1	1	
Preterm^a^	39	10 (25.6%)	4.21 [2.10 to 8.42]	3.11 [1.12 to 8.65]	0.03

^a^ Preterm = Born alive before 37 completed weeks of gestation.

After model adjustment, however, only maternal age, birth weight and infant maturity at birth remained independently associated with HIV infection or death of the infant. Children of women <25 years of age were more likely to be HIV‐positive or die compared to children of women ≥25 years (Hazard ratio (HR) = 2.51, 95% CI: 1.29 to 4.87). Among infants born to adolescent and young women (14‐24 years), 25 (14.4%) were infected or died compared to 27 (5.8%) infants born to older women (25 to 45 years).

For every 100‐gram increase in birth weight, the risk of HIV infection or death in infants decreased by 7% (HR = 0.93, 95% CI: 0.86 to 0.99); and preterm infants had a higher risk of infection or death compared to term infants (HR = 3.11, 95% CI 1.12 to 8.65).

## 4 DISCUSSION

Our results show that mother‐to‐child transmission of HIV by 24 months of age is 2.9% and HIV‐free survival among HEIs in the public healthcare setting of Lesotho is 91.8%. These results demonstrate the effectiveness of universal, lifelong ART implemented among breastfeeding communities residing in a high HIV‐prevalence setting, and are consistent with findings of a similar study conducted in urban Kigali, Rwanda between 2013 and 2016 in which Gill et al reported a 24‐month HIV transmission of 2.2% and an HIV‐free survival rate of 93.2% [7].

Several studies conducted within sub‐Saharan Africa have assessed HIV‐free survival up to six weeks of infant age [9, 10, 11, 13]. Outcomes at six weeks of infant age reflect the transmission that occurs during pregnancy and the peripartum period but fail to show the outcomes of continued HIV exposure that occurs during the breastfeeding period. In many sub‐Saharan African settings, women breastfeed their babies throughout the first year of life and often through the second year [13]. The results of our study are noteworthy since they cover the entire breastfeeding period and take into consideration the diverse infant feeding practices. Mixed feeding, though practised by a number of women in our cohort, was not associated with HIV transmission. A community‐based cohort study conducted in rural Zambia reported an HIV‐free survival of 96.3% at 12 months of infant age, slightly higher than found in this study [15]. In Zimbabwe, serial cross‐sectional community‐based sero‐surveys that evaluated the PMTCT effectiveness at nine to eighteen months of infant age, showed a 10% MTCT rate and HIV‐free survival of 89.6% in 2012, with a 2014 repeat survey showing a reduction in MTCT to 4.8% and an increase in HIV‐free survival to 95.1% with the WHO Option A PMTCT strategy [16].

For PMTCT programmes to be effective in reducing infection and death among infants, a “cascade” of PMTCT services needs to be effectively delivered and accessed during antenatal care, delivery, and in the postnatal period. These services include mother and partner HIV testing and counselling, and if HIV positive, initiation and retention on ART; prompt initiation of antiretroviral prophylaxis for the HEI once born, and infant HIV testing at six weeks of age and subsequent time points if HIV negative until six weeks after discontinuation of breastfeeding [17, 18]. In addition, prevention counselling and repeat tests need to be carried out on HIV‐negative mothers with immediate initiation of ART whenever HIV is diagnosed and testing of the child if breastfed.

Due to challenges in the healthcare system in many sub‐Saharan African countries like Lesotho, these proven interventions are seldom fully implemented. In many settings, only a proportion of women receives ANC at health facilities and these services are often sought late in pregnancy or at delivery [19]. The results reported here clearly demonstrate that the implementation of universal maternal ART markedly reduces MTCT even in non‐ideal public health settings where ANC and postnatal services are suboptimal.

For the HEI, prevention of HIV transmission from the mother largely depends on the mother’s ability and willingness to initiate and continue with lifelong ART and on her adherence to treatment to maintain sustained virological suppression. When mothers do not adhere to treatment or become lost to follow‐up, treatment interruptions occur that lead to an increase in the risk of HIV transmission to the infant [20, 21, 22]. In addition, HIV‐negative mothers who acquire infection during pregnancy risk transmitting the virus to their infants. Acute HIV infection during pregnancy or breastfeeding markedly increases the risk of MTCT of HIV [23, 24, 25, 26]. We found that HIV transmission was four times higher in HIV‐negative women who seroconverted compared to women who were HIV positive at delivery. This underscores the need for additional protections for HIV negative women during pregnancy and the breastfeeding period. Nevertheless, the majority of the transmissions still occurred among women who were known to be HIV positive at enrolment. The suboptimal viral suppression seen in some of these women may have contributed to the transmission. Although not assessed, viral resistance to ART may have led to the observed viraemia.

The significantly lower survival of HEIs compared to HUIs in this study draws attention to the continued need for early diagnosis followed by prompt initiation of ART for all HIV‐positive children and close follow‐up of all HIV‐exposed uninfected (HEU) children. These HEU infants and children have in previous studies been found to have impaired growth and higher infectious disease morbidity than their HIV‐unexposed counterparts [27, 28, 29]. The underlying mechanisms leading to worse adverse outcomes in these group of HEI are not fully known, but are likely to be a combination of several factors including intrauterine growth retardation and preterm birth, seen more often among HIV‐exposed newborns [30], immune dysfunction [31] and other adverse environmental and socio‐economic conditions.

Another important finding is the observed association between maternal age and HIV infection or death in infants. A significantly higher proportion of infants born to adolescent or young women was infected or died compared to the deaths or infections seen in infants of older women. As the universal ART programme in Lesotho continues to mature, there is a need to explore the underlying factors contributing to the negative PMTCT outcomes seen among adolescent girls and young women. A large study in Zimbabwe found that adolescent pregnant women were less likely to know their HIV status or be on ART prior to ANC attendance, which could be one of the contributing factors [32]. To improve child survival, there is a need to reinforce current MCH and PMTCT efforts with appropriate interventions that take into consideration the increased vulnerabilities seen among these young mothers. In addition, these results highlight the need to strengthen sexual and reproductive health services for young women to prevent HIV acquisition and pregnancy.

Our study had some limitations. We were only able to assess HIV‐free survival among infants born to women who attended ANC services at health facilities. It is likely that some pregnant women did not attend ANC or delivered at home without visiting the facilities leading to an underestimation of our study outcomes. We are therefore unable to estimate HIV‐free survival at the population level. In addition, children who were lost to follow‐up may have been infected or died leading to underestimation of these outcomes. Information on diagnoses preceding death of infants was, in some cases, obtained from caregivers through phone interviews or abstracted from patient records at the health facilities. The accuracy of abstracted data depended on the information recorded by the clinicians who saw the patients. It is possible that some of the diagnoses were inaccurate. Post‐mortem examination of deceased infants was not conducted, making it difficult to determine the cause of death. A number of women had suboptimal viral suppression and our data on adherence to ART had gaps and quality issues. We were unable to use it in this analysis, and this may have limited our understanding of the reasons for the high viral load seen. Despite these limitations, our study provides promising results of PMTCT implementation from a region with the highest HIV prevalence in the world.

## 5 CONCLUSIONS

The implementation of universal ART in the Lesotho’s public health system has markedly reduced HIV MTCT rates that approached the global target of <2%. However, despite access to ART, HIV‐positive children still had lower survival than their HIV‐negative counterparts, as did HIV exposed but uninfected children. The results here provide additional evidence on the effectiveness of current PMTCT strategies and underscore the need for differentiated PMTCT interventions that address the additional vulnerabilities encountered by infants of adolescent and young women.

## COMPETING INTERESTS

The authors do not have any competing interests.

## AUTHORS’ CONTRIBUTIONS

Funding acquisition: AT, MGG, AI, SGK, LG. Conceptualization and initial protocol development: RM, MGG, AT, AI, MN, MT, SN, ML, SGK and LG. Protocol revisions and refinement of data collection tools: VT, RM, MGG, AT, MM, MN, MT, SN, SGK and LG. Investigation: VT, RM, MGG, AT, AI, MN, MT, SN, ML, SGK and LG. Project administration and Data Collection: VT, RM, MGG, AT, MM, SGK and LG. Supervision: VT, RM, MGG, AT, MM, MT, SN, ML, SGK and LG. Drafting the manuscript: VT. Review and editing of manuscript: VT, RM, MGG, AT, MM, AI, MN, MT, SN, ML, SGK and LG.
